# Long-Term Effect of Interactive Online Dietician Weight Loss Advice in General Practice (LIVA) Protocol for a Randomized Controlled Trial

**DOI:** 10.1155/2014/245347

**Published:** 2014-03-30

**Authors:** Carl J. Brandt, Vibeke Brandt, Mathilde Pedersen, Dorte Glintborg, Søren Toubro, Jesper Bo Nielsen, Gunther Eysenbach, Kirsten Brandt, Jens Søndergaard

**Affiliations:** ^1^Research Unit for General Practice, Institute of Public Health, University of Southern Denmark, J. B. Winsloews Vej 9A, 5000 Odense, Denmark; ^2^Department of Endocrinology M, Odense University Hospital, Kløvervænget 64, 5000 Odense C, Denmark; ^3^Reduce - A Research Unit, Tempelvej 24, 4390 Vipperoed, Denmark; ^4^Institute of Health Policy, Management and Evaluation (IHPME), University of Toronto, Canada M5T 3M; ^5^Human Nutrition Research Centre, School of Agriculture, Food & Rural Development, Newcastle University, Agriculture Building, Kings Road, Newcastle upon Tyne NE1 7RU, UK

## Abstract

*Background.* Internet-based complex interventions aiming to promote weight loss and optimize healthy behaviors have attracted much attention. However, evidence for effect is lacking. Obesity is a growing problem, resulting in an increasing demand for cost efficient weight loss programs suitable for use on a large scale, for example, as part of standard primary care. In a previous pilot project by Brandt et al. (2011) without a control group, we examined the effects of online dietician counseling and found an average weight loss of 7.0 kg (95% CI: 4.6 to 9.3 kg) after 20 months. *Aims and Methods.* To analyze the effects of a complex intervention using trained dieticians in a general practice setting combined with internet-based interactive and personalized weight management support compared with conventional advice with a noninteractive internet support as placebo treatment in 340 overweight patients during a 2-year period. Primary endpoints are weight loss and lowering of cholesterol (LDL). We will also explore patients' sociodemographics and use of the intervention as well as the health professionals' views and perceptions of the intervention (their role and the advice and support that they provide). *Perspective.* The project will generate knowledge on the cost-effectiveness of a complex internet-based intervention in a general practice setting and on barriers and acceptability among professionals and patients.

## 1. Introduction

Obesity is a growing problem and more than 30% of the European population is obese with a body mass index (BMI) >30 kg/m^2^. Weight loss decreases the risk of obesity related complications such as type 2 diabetes, cardiovascular events, and joint and musculoskeletal diseases as well as improves fertility and reduces the risk of spontaneous abortion. The expected economic implications of weight loss among the obese are reduced sick leave and substantially lower demand on health support.

Controlling dietary intake and increasing physical activity through behavioral change programs in a face-to-face setting have previously been demonstrated to produce significant weight loss [1]. However, implementing such programs into general practice has shown to be difficult due to a number of barriers [2]. Face-to-face consultations are time consuming both for the health professionals and for the patient, and many general practitioners do not offer sufficiently extensive behavioral support to fully achieve the potential effect [2]. In order to achieve a major behavioral change, information must be so clearly relevant for the patient that they cannot easily choose to ignore it [3]. When face-to-face consultations are used on their own (without extensive follow-up measures), long-term effects compared to controls are difficult to detect [4]. Furthermore, in Denmark access to professional dieticians is limited due to the costs of the public health care system.

New interactive media can be used in weight management [5–12]. The effects in primary care have been variable [12], but using the internet seems to be more cost-effective, even when the magnitude of the effect is not affected [13]. Personal individual advice can be delivered either synchronously or asynchronously. By delivering the advice asynchronously important communication can be managed at a very low cost due to the fact that digital communication is stored and parts of it can be reused for many patients in templates or articles [14]. Matched peer support through online communities where patients can meet other patients in a similar situation as themselves can offer important support, and established relations between patients and health professionals seem to be important drivers for successful outcomes [6, 12, 15].

In a recent prospective pilot study without a control group we investigated the effect of a medium intensive online dietician advice combined with a community using the existing commercial online weight loss management program “SlankeDoktor” [16]. We found a mean weight loss from baseline of 7.0 kg (95% CI : 4.6–9.3 kg) after a mean maintenance period of 20 months among 21 patients with an initial BMI of 36.4 kg/m^2^ [17].

## 2. Aims

- To evaluate the impact of a complex intervention using face-to-face contact with trained dieticians combined with interactive online support and follow-up, as compared with usual care, on BMI and metabolic risk factors.
- To explore the feasibility of the intervention by assessing patients' sociodemographic characteristics and use of the different services in the intervention in relation to their weight loss.
- To explore the usability and applicability of the intervention in relation to the health professionals' view and perceptions of the intervention (their role and the advice and the support that they provide).

## 3. Materials and Methods

A multicentered two-arm randomized controlled trial.

### 3.1. Patients

**Figure 1:**
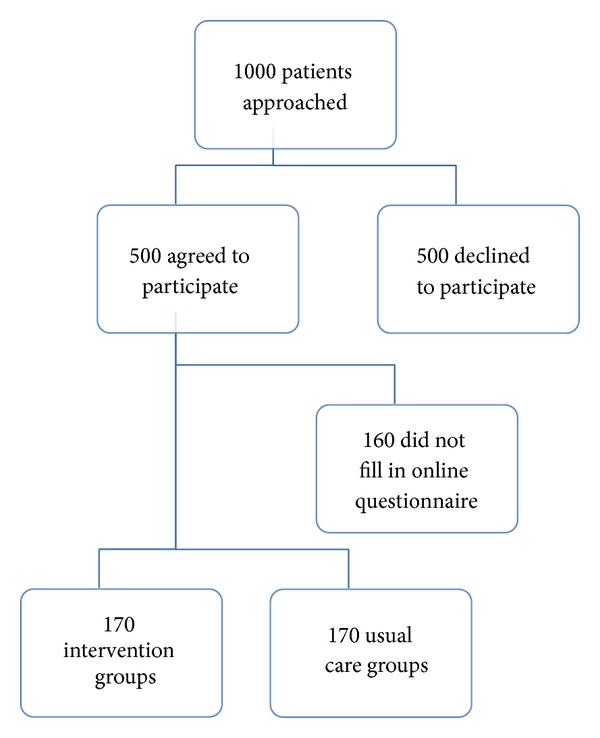
Flowchart for patients.

The study will recruit 340 patients registered in general practices within 4–6 medical centers, representing a population of approximately 40,000 citizens. The medical centers will be invited to participate and consecutively included. They will be both rural and city centers and have between 2 and 10 doctors working at each center. Doctors and practice staff will hand out information material about the study. Patients are included consecutively until there is a total number of 340 patients randomized to either intervention group or control group, respectively. The practice staff will register everyone agreeing to participate. We expect to include initial 500 patients before randomization as our experience from the pilot study is that 25% of those who agree to participate do not fill out the online registration form required to be randomized [17] (Figure 1).

### 3.2. Inclusion Criteria

- The patient has a BMI ≥ 30 kg/m^2^.
- The patient is 18−70 years old.
- The patient provides informed consent.
- The patient has home access to the internet.
- The patient is not pregnant.
- The patient has no serious or life threatening disease.
- The patient has no weight loss >3 kg within the last 2 months.

### 3.3. Exclusion Criteria

- The patient fails to complete the initial questionnaire online.
- The patient becomes pregnant during the study.
- The patient experiences serious or life threatening disease during the study.
- The patient moves to another GP practice not participating in the study.
- The patient has a serious eating disorder or develops one during the study (e.g., rapid weight changes).

Patients will be invited consecutively by doctors and practice staff. The first information is received at the medical center, ensuring full confidentiality. Patients who receive information concerning the study from doctors or practice staff and who agree to participate are registered and scheduled for an appointment with a nurse. The patients will be able to bring a friend or relative in accordance with ethical guidelines for biomedical experiments with humans. An appointment with a nurse is scheduled within 7–14 days after the information; thus the patient has sufficient time to reflect on the decision to participate. The nurse obtains the approved informed consent and proceeds with weighing and measuring the patient. The patient is then instructed to use a web link to a standard quality of life questionnaire including SF12 and other relevant questions from the webpage www.praksis.sundhedsdoktor.dk. Patients are randomized immediately after the questionnaire is filled out, using an automated computer algorithm. This procedure ensures that dropouts can be recorded as well as certain characteristics for the patients who choose to drop out.

### 3.4. Intervention Group

**Figure 2:**
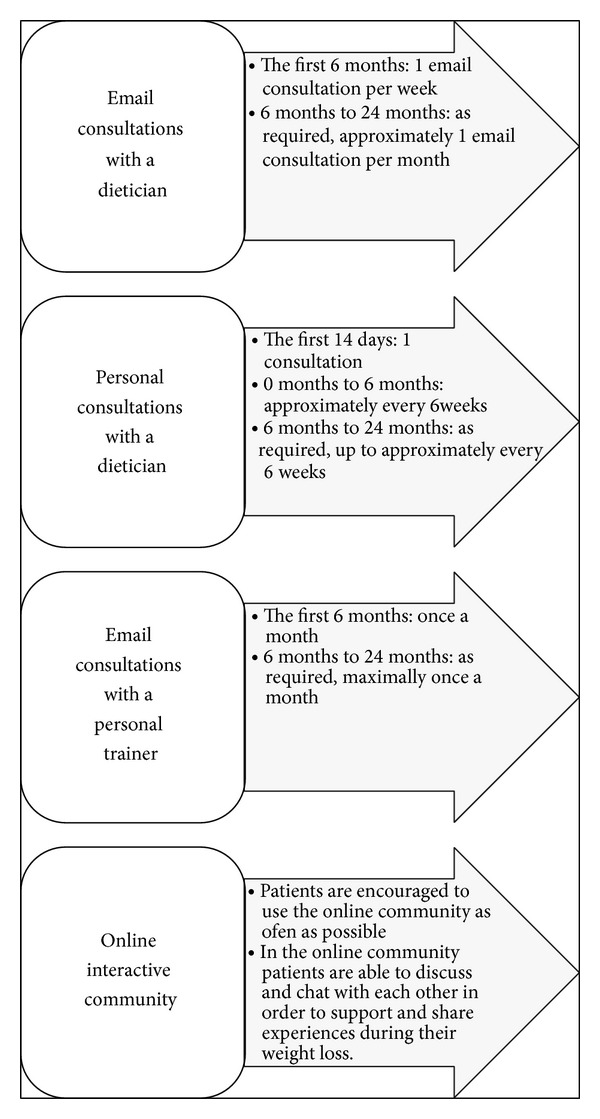
Consultation schedules in the intervention group.

Patients randomized to the intervention group receive a login to the website “http://www.praksis.sundhedsdoktor.dk/” as well as a consultation with a certified clinical dietician within 2 weeks. The website has been developed over a 12-year period with focus groups, usability testing, and user involvement from both patients and health care professionals. At “http://www.praksis.sundhedsdoktor.dk/” the patients fill in a daily diet record as well as their comments, concerns, and questions to the dietician, who will have access to all patient profiles. Patients can monitor and follow their weight loss progress at the website. They have access to a community where they can discuss and chat with other patients and share weight loss experiences as well as give advice and support to each other. Patients receive subsequent 15–30 min consultations with the dietician by appointment at the start and up to every 6 weeks for 6 months to a total of 5 (Figure 2). The certified clinical dieticians also provide individual asynchronous online consultation [14] according to their patients' needs and based on information supplied via the website. The online consultations are weekly during the first 6 months and after that they are monthly. The dietician generates a daily caloric reduction plan of approximately 2400–3200 KJ based on the patients' starting weight, diet record, and physical activity. The dietary advice will be as standardized as possible. In collaboration with the patient goals for obesogenic behavior changes are set up like “eat breakfast every day,” “no late night meal or snack,” and so forth that follow the dietary guidelines given at “http://www.praksis.sundhedsdoktor.dk/” and The Danish Health and Medicine Authority (Sundhedsstyrelsen). A personal trainer evaluates the exercise by monitoring information given by the patients and encourages them to increase their physical activity to a level of at least. Patients will be encouraged to increase their physical activity to a level with at least 30 min daily. Three times a week the physical activity is suggested to be of moderate to high intensity.

### 3.5. Control Group

Patients randomized to the control group will receive usual treatment through the medical center, which consists of approximately monthly face-to-face consultations with a nurse or doctor. Additionally they will receive a “placebo treatment” in the form of a login to a website that looks like http://www.praksis.sundhedsdoktor.dk/, but contains only general non-interactive and non-personalized dietary and exercise advice (e.g. “all about diet” from the National Food Agency (Fødevarestyrelsen)), but with neither dietician consultations nor community membership.

### 3.6. Data

Answers to the standard quality of life questionnaire are collected at the medical center by a nurse at inclusion at 6 months and at 24 months.

Patient characteristics will be entered at the start. Patient height, waist and hip circumference, blood lipids, Hba1c, and weight are measured at inclusion after 6 months and 24 months.

The blood samples will be collected by the bioanalyst in the practice in accordance with guidelines for the handling of blood measurements in Danish general practice and analyzed at the certified laboratory at Odense University Hospital.

Data will be continuously added to a database by the patient, nurse, and dietician.

Number of logins, number of entrances in the different sections of the site, and user patterns will be collected from the database at 6 months and again at 24 months. Dieticians, nurses, doctors, and other health professionals from the medical center will be interviewed about the intervention in relation to their work, their role, their perception of the treatment offered, and the strength and weaknesses seen from their perspective in accordance with the MAST models organizational dimension (Table 1) [18].

In the case of exclusion before the end of the trial, for example, by pregnancy, the patient will if feasible be asked to complete a final questionnaire and have objective parameters measured in order to provide data for an “intention to treat” analysis. The medical centers will be responsible for this. Data and answers to questionnaires will be collected at 6 months and 24 months by independent observers that will assign each patient to a number to mask the identity for the analysts.

## 4. Statistics

The primary objective of this study is measurement of changes in body weight and waist circumference. Weight loss in the intervention group and control group will be analyzed using appropriate statistical procedures. A weight loss of 5–7 kg in the study group and 2-3 kg in the control group is expected after 6 months of treatment. After 2 years we expect the control group to return to the starting weight, while the study group is expected to maintain a weight loss of 3–5 kg. A power calculation, based on standard deviations observed in a previous pilot project [17], shows that to detect a difference in weight loss of 2.5 kg with a power of 90% requires 142 patients per group. 170 patients will be randomized to each group to allow for dropouts. Secondary outcome such as logins, web site activity, and user patterns will be analyzed in relation to the different quartiles. The most active quartile will be compared to the least active quartile in relation to weight loss, and other measured parameters. If the difference in sustained weight loss after 2 years is greater than 2.5 kg, the planned total of 340 patients will allow analyses of interactions between treatment, gender, age and obesity level. Due to this, the randomization will be stratified by gender, while potential effects of and interactions with other recorded characteristics such as smoking, age and obesity level will be analyzed as continuous or discrete variables as appropriate.

All data will be blinded and the randomization code and identity of the patients will not be revealed to the data manager before analyses are completed. Data will be analyzed by statistical experts. After the study, all data will be made accessible on the internet in anonymized form to allow full peer scrutiny and facilitate secondary research.

## 5. Discussion

The intervention to be assessed in the present study has been developed since 2002 by a team of dieticians, computer programmers, and physicians as the commercial weight management program “SlankeDoktor” [16]. The guiding principle was to improve the cost-effectiveness of established best practice for dietician-supported weight management by using the internet to facilitate interactive communication between the dieticians and the users as well as among the users (peer support through online community). This program was developed iteratively from an initial prototype through trial and error to what is now a well-established commercial product used by 10.000 predominantly healthy overweight or moderately obese users in 2013. Regarding the use in general practice for treatment of obese patients, this program has been tried on a pilot scale with good results [17]. However an appropriately designed randomized controlled trial is required in order to assess the actual cost-effectiveness of this approach compared with other options.

In the Danish health care system, general practitioners are obliged to identify and offer appropriate support to obese patients whose health would benefit from a weight loss, and a prespecified sum per patient is available to finance this support. In most cases this support (“usual care”) consists of 4–6 monthly face-to-face consultations with a health professional, usually a nurse or physician, which includes provision of general weight management information such as brochures and educational internet sites. So the present study has been designed to allow direct comparison between the intervention (dietician-supported internet community) and a single-blind control treatment. The two arms provide the same number of face-to-face consultations which include the sessions to measure and sample the patients. The usual care control treatment will be provided in a way so the patients are not aware which arm they are on by using a placebo website that superficially resembles the interactive treatment without providing any personalized support. This study will utilize the Danish general practice setting, where it is possible to test the combination of face-to-face consultations with dieticians (establishing the relation), real-time online reporting, life style advice, and community peer support as a full scale trial. The online tool in this study will be integrated into the exiting electronic journal system of the general practitioners to emulate how it would be implemented if it was part of the standard offer.

Additional information will be collected from patients and health professionals as feedback to enable further improvements, primarily regarding how to optimize the experience for both patients and health professionals as well as how to identify which patients are most likely to benefit from this type of intervention, in order to optimize resource allocation to this and other options for weight management.

A potential limitation to the chosen study design is that the number of doctors recruited is limited and those who take part might be more interested in new technologies than the average general practitioner. Another weakness of the present study is that the intervention comprises several dimensions of complexity, which preclude evaluation of each aspect of the intervention separately. However, with this new technology doctors will be able to prescribe exercise and diets and obtain insight into patient performance at a much more detailed level than seen before. Compared with other published studies such as [6] the study is a long-term study without monetary incentives for patients, is designed to work in a routine primary care setting, and takes into account the value chain established in the Danish health care system. If the study is successful, this will make it easy to implement the program nationwide and internationally for the benefit of many overweight patients. Additionally the organizational learning provided by this study will be of importance for implementing other medical online support systems in the future and providing new opportunities for the handling of patients in primary care with modern technology and patient empowerment.

## Figures and Tables

**Table 1:** Example of organizational outcome measures from the MAST model.

	*Staff, training, and resources *
*√*	Changes in distribution of work (working hours spent) between the professions involved (task shifting)
*√*	Changes in staff requirements (reduction or increase of working hours) for each profession involved
*√*	Time spent by members of staff on training in order to learn to apply telemedicine devices
*√*	Changes in hours spent on various procedures in clinical pathways, measured for each relevant profession
	*Interaction and communication *
*√*	Amount of electronic communication
*√*	Changes in information and reporting system
*√*	Changes in number of face-to-face patient consultations
*√*	Changes in the way medical staff communicate
*√*	Changes in the way the medical staff work together (generalists/specialists, doctors/nurses, etc.)
	*Structure outcomes *
*√*	Changes in the number of units offering treatment
*√*	Number of organisational units set up especially for telemedicine (if any)
*√*	Changes in the organisation of generalist and specialist tasks
*√*	Changes in geographical spread
*√*	Time spent on travel, staff
*√*	Time spent on travel, patients
	*Culture outcomes *
*√*	Staff attitudes towards telemedicine applications
*√*	Staff experience with the use of telemedicine applications
